# Energy Aware Load Balancing Framework for Smart Grid Using Cloud and Fog Computing

**DOI:** 10.3390/s23073488

**Published:** 2023-03-27

**Authors:** Saurabh Singhal, Senthil Athithan, Madani Abdu Alomar, Rakesh Kumar, Bhisham Sharma, Gautam Srivastava, Jerry Chun-Wei Lin

**Affiliations:** 1 Department of Computer Engineering and Applications, GLA University, Mathura 281406, Uttar Pradesh, India; 2 Department of Computer Science and Engineering, Koneru Lakshmaiah Education Foundation, Vaddeswaram 522302, Andhra Pradesh, India; 3 Department of Industrial Engineering, Faculty of Engineering-Rabigh, King Abdulaziz University, Jeddah 21589, Saudi Arabia; 4 Chitkara University Institute of Engineering and Technology, Chitkara University, Rajpura 140401, Punjab, India; 5 Department of Mathematics and Computer Science, Brandon University, Brandon, MB R7A 6A9, Canada; 6 Department of Computer Science and Mathematics, Lebanese American University, Beirut 1102, Lebanon; 7 Research Centre for Interneural Computing, China Medical University, Taichung 40402, Taiwan; 8 Department of Computer Science, Electrical Engineering and Mathematical Sciences, Western Norway University of Applied Sciences, 5063 Bergen, Norway

**Keywords:** cloud computing, energy consumption, fog computing, load balancing, resource utilization, smart grid

## Abstract

Data centers are producing a lot of data as cloud-based smart grids replace traditional grids. The number of automated systems has increased rapidly, which in turn necessitates the rise of cloud computing. Cloud computing helps enterprises offer services cheaply and efficiently. Despite the challenges of managing resources, longer response plus processing time, and higher energy consumption, more people are using cloud computing. Fog computing extends cloud computing. It adds cloud services that minimize traffic, increase security, and speed up processes. Cloud and fog computing help smart grids save energy by aggregating and distributing the submitted requests. The paper discusses a load-balancing approach in Smart Grid using Rock Hyrax Optimization (RHO) to optimize response time and energy consumption. The proposed algorithm assigns tasks to virtual machines for execution and shuts off unused virtual machines, reducing the energy consumed by virtual machines. The proposed model is implemented on the CloudAnalyst simulator, and the results demonstrate that the proposed method has a better and quicker response time with lower energy requirements as compared with both static and dynamic algorithms. The suggested algorithm reduces processing time by 26%, response time by 15%, energy consumption by 29%, cost by 6%, and delay by 14%.

## 1. Introduction

Smart Grid (SG) is a tool that electric power companies use to keep track of and control how much energy their customers use. Smart meters that can connect in both directions allow for better energy monitoring [1]. Load balancing is a part of SG’s distributed energy management system that makes communication more secure and reliable. Businesses can manage their power systems better if they keep track of how much energy they use. The SG energy burden can be changed and maintained [2]. Control centers keep an eye on the power supply and safety measures to make sure that the load-balancing system used by SG for communication stays in good shape.

With more smart devices, more space for storage and security is required. Cloud computing can resolve these problems. In the past few years, cloud computing has become more and more popular. Cloud computing makes it possible to use online services [3] since it is fast, easy to change, and cheap [4,5]. Cloud data centers are where most Physical Machines (PMs) are kept. Thanks to virtualization, cloud service providers can now make it easier for people to share resources and virtual machines (VMs). With task scheduling, the sequence of execution of tasks can be obtained, with the recent focus being on energy conservation to minimize cost and other QoS parameters. Ghafari et al. [6] have discussed various task-scheduling algorithms by categorizing them into three categories. A multi-objective load-balancing algorithm combining the features of Firefly and PSO [7]. The algorithm selects the best value of the global parameter with a small distance in which PSO is used to enrich response while Firefly is used to control the search space. 

In a smart grid, cloud computing connects power company-owned substations and power plants. Built-in redundancy improves reliability, security, and resiliency [8,9]. Because data are abundant, SG applications require scalable systems. Peak hours demand more resources than off-peak hours, so resource needs vary throughout the day. SG architectures require scalability, elasticity, security, robustness, and sharing of resources [10]. Fog computing was coined in 2014 [11]. Fog computing provides low-latency services at the network’s edge. Fog computing reduces cloud congestion and improves user communication since the WiFi is used to connect users and fog nodes. Fog computing provides services nearby when it receives user requests, and the VM’s many applications work together to fulfill them [12]. Users and fog nodes need network resources to communicate, thus network resource demand hinders communication. 

Working with cloud and fog nodes in an SG environment raises many concurrent issues. These requests could be made for services such as power or other necessary commodities. Through the utilization of cloud and fog nodes, it is possible to create an SG network link between cloud environments and SG users. The user expresses their requests to the fog nodes, which are relayed to the cloud [13]. In the scenario, the user approaches the fog nodes with a request for electricity, and the fog nodes react by providing the requested services. The nearest available resource that can handle such requests provides these services.

Modern metering infrastructure connects smart meters to the advanced metering infrastructure (AMI). The infrastructure uses non-standard communication technology. The AMI applications and service providers are summarized in Table 1. The nodes are responsible for discovering and confirming any additional nodes or resources if there is not a central server acting as command and control, making privacy, trust, and authentication more difficult.

The scenario consists of three layers. The top layer is a centralized cloud. In the second level, fog nodes are present, with each fog node including several virtual machines that consume the least amount of energy. The buildings are organized into groupings that are subsequently subdivided into clusters. There is a close relationship between the fog nodes in that area and every PM. From these aggregations of structures, fogs get requests from the users. The data is stored in the fog for a brief amount of time before being sent to the cloud, where they remain permanently. The major purpose is to offer people the services they require in the shortest feasible time. Response Time (RT) describes the amount of time required for the fog node to respond to a request [14]. The duration of time before a request is processed by the fog node is termed the Processing Time (PT). If the fog node is already at capacity, the request will not be handled for an extended time. On the other hand, it will take less time to complete jobs if there is not enough movement in the fog nodes. In the developed model, a metaheuristic algorithm called Rock Hyrax optimization [15] is employed to evaluate fog nodes for being overutilized and underutilized.

### 1.1. Problem Statement

Cloud data centers have several PMs [2]. Cloud service providers can pool resources and offer virtual machines using virtualization. Cloud service providers may bundle multiple VMs into a few PMs to save on operational expenses and energy consumption. Grid computing is unsuitable for the SG environment because it only allows for one-way interaction. For such applications, fog collects requests to minimize latency and cloud computing. They surpass each other’s shortcomings. The fog has increased availability and latency, while the cloud has high computing. SG may cut off or shift electricity during peak hours. Real-time electricity request management helps service providers serve clients better. Local servers can deliver data quickly but cannot manage random and large queries. Balancing cloud node loads ensures that no node is overloaded and allows us to turn off less-used nodes to save energy.

The problem is represented as a Multidimensional Multiple-choice Knapsack (MMKP) Problem. MMKP is an NP-hard problem. Consequently, it is not always possible to discover a practical solution in a reasonable amount of time, especially when the issue at hand is significant. For this, a nature-inspired metaheuristic algorithm called Rock Hyrax Optimization has been used. The Rock Hyrax are small mammals found in Africa that mimic the divide-and-conquer strategy while foraging. The proposed algorithm RHO comprises different parts. Initially, RHO detects underutilized and overutilized fog nodes. The algorithm moves the underutilized virtual machine to overutilized nodes if a node is found to be underutilized during the process. The system considers all hosts other than the overutilized host as underutilized hosts. The intention is to transfer the underutilized virtual machines and put such hosts into sleep mode. The transfer of underutilized virtual machines is done to minimize energy consumption because if underutilized virtual machines are active; they will continue to consume the same amount of energy even when not processing any requests. By shifting underutilized VMs into sleep mode and relocating the virtual machines to overutilized PM, power consumption is reduced.

### 1.2. Motivation

Cloud and fog computing are both used in smart buildings to distribute resources more effectively. The major goal of the proposed work is to save consumer costs while also lowering energy usage. With more users using cloud services, the energy required to maintain the service and the number of requests made to cloud data centers also increase. As a result, it is crucial to manage the energy infrastructure and address customer needs. RHO is used in this work to balance off the load. It is challenging to manage the requests of several users simultaneously. Therefore, clouds and VMs were connected with fog. The VM of the user issued a command into the fog. Some fogs may be underutilized, while others remain overutilized. By shifting underutilized fogs into sleep mode and relocating their virtual machines to overutilized fogs, power consumption is reduced.

### 1.3. Contribution

The main contributions of this paper are as follows:

- A nature-inspired load balancing algorithm for a smart Grid environment linked with cloud and Fog.
- Using a meta-heuristic approach for proposing a multi-objective load balancing optimization algorithm.
- Minimization of response time and energy consumption is accomplished with better resource utilization

The rest of the paper is organized as follows. Section 2 summarizes related work. The proposal with mathematical formulation and framework is presented in Section 3. Section 4 elaborates on the results obtained after the implementation of the proposal, and finally, the conclusion is summarized in Section 5.

## 2. Related Work

Various studies have been done on how SG applications could use the cloud to improve their dependability and performance. Fatima et al. [16] suggested that a good way to share resources in residential areas would be to combine a cloud-based environment with a fog-based environment. In SG [17], Particle Swarm Optimization (PSO) is used in a cloud-fog-based model for load balancing to make the best use of the available resources. By combining an enhanced Cuckoo Optimization Algorithm with PSO, Bouyer et al. [18] provide a hybrid technique for load balancing. Javaid et al. [19] used a cloud-fog model to improve resource allocation in smart buildings. Mareli et al. address the cost-effectiveness analysis for modifying settings [20] and are primarily concerned with switching and moving resources to meet requirements. 

For balancing load in a smart grid, an artificial bee colony algorithm is used in [21]. For balancing the load, Mohanty et al. [22] use a gradient-based optimizer. A hybrid algorithm is proposed in [23] by combining whale optimization and bat optimization for balancing load in a smart grid environment. To minimize energy in a virtualized environment, Sharma et al. [24] embedded the system with the green computing principle. For IoT-based devices, a comparative analysis is presented in [25] while [26] discussed the security of IoT devices using blockchain. Kumar et al. [27] used the BAT algorithm for managing load in the cloud environment, while cuckoo and artificial bee colonies were combined for managing the load on the cloud [28]. 

Abualigah et al. [29] presented a new metaheuristic algorithm based on the hunting behavior of a prey Aquila. Four different methods are presented to show the hunting behavior of the Aquila. A new metaheuristic approach based on prey–predator interaction is explored [30]. The approach works by considering body mass and interaction. Local search is carried out by the predator having the best energy gain, while others are in further exploration. Using a binary PSO, Akram et al. [31] have allocated resources in a load-balancing manner. Inertia weights are used to restrict the search space.

Singhal et al. [32] discuss mutative Ant Colony Optimization in the cloud environment for balancing the load on the servers. Security is also a challenge for the smart grid. Unexpectedly, clients’ privacy is affected by smart meters. Data about how a customer uses energy are stored at the meter. Large-scale smart meter misuse might have grave consequences. Incorrect use of data in control systems might damage electrical infrastructure. Many studies have tried to address the issue of security in Smart Grid. Kondoro et al. [33] investigated the security protocols in IoT communication for real-time properties to determine the impact of security on the Smart grid. To ensure security in the Fog environment, Verma et al. [34] used Elliptic Curve Cryptography for authorization, and Dhaou et al. [35] discussed security in a smart grid environment when communication is happening. A review of centralized security schemes is presented [36] for an IoT environment, carrying out a comparison over several factors like network model and architecture type. In addition, various issues and challenges concerning fog networks were discussed [37] while focusing on IoT applications. A summary of the reviewed paper is presented in Table 2.

## 3. System Model

**Figure 1:**
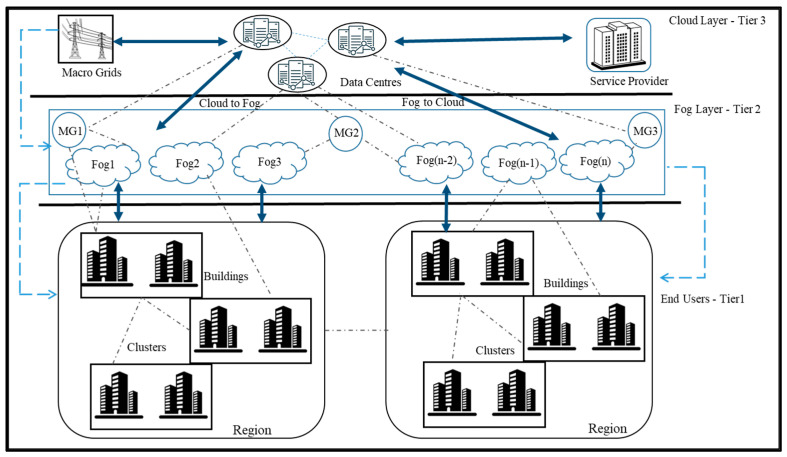
Proposed system model.

Cloud service providers use global data centers for computing and storage. The suggested system paradigm for a cloud–fog operational environment incorporates several fog data centers and cloud data centers. Figure 1 shows the proposed three-layer system model. At the bottom of the model is the end-user layer where SG applications are running, the middle layer is the fog node layer; and the top layer is the cloud layer which houses data centers and services provided by service providers.

A system model with three tiers is proposed, where tiers are linked together so that data can flow between them. The end-user layer has N buildings, each with several residential units. Every building needs an energy storage system and a renewable energy generator. Because it uses renewable energy sources, it is therefore substantially better for the environment than others. Similarly, during low-generation hours, excess energy is retained in a storage system to satisfy the load requirement in the future.

A fog node consists of numerous hardware and software components. The fog node adds another layer of protection and is responsible for managing requests from users in all locations of the world. The requests involve both information access and electricity requirements. The implementation of the fog is intended to minimize the Cloud’s load. The fog node layer stores the data temporarily before being transmitted to the cloud for permanent storage. End consumers can benefit from low-latency services using this strategy. In the cloud, virtual machines are accountable for handling user requests. The user can send its request for energy to the Micro Grid (MG), which is in turn connected to fog nodes. 

The fog layer can receive all transmissions regarding the production, consumption, and scheduling of appliances inside a home’s energy system. The layer runs its application using various cloud services. The building has smart meters connected to fog devices. Because of the cloud–fog environment, all families can share information regarding power shortages and surpluses. Connecting smart meters to a network enables communication with one another. The SG supports multiple wireless connection choices, including Wi-Fi.

The fog layer is composed of multiple distinct fog nodes where every intelligent structure is connected to a fog device which is made up of resources in the form of virtualized hardware such as memory, and storage. Utilizing virtualization, the fog node may monitor and manage multiple VMs housed on a single physical computer. The Virtual Machine Manager (VMM) enables the coexistence of several operating systems on a single hardware platform. The VMM or hypervisor acts as an interface between guest operating systems and virtual machines running on top of it. Many different applications can be run on each VM. The user is connected to the Cloud via a communication layer known as the Fog. 

The lowest layer is the core cloud layer which houses data centers to provide the processing power and storage required by consumers. Based on applications and requirements, the cost is decided on a pay-per-use basis. The cloud layer consists of several servers that perform processing on demand. The relationship between clouds and fog nodes is immutable. 

User data are temporarily stored in fog nodes before being transferred to the Cloud in response to a request for permanent storage. The computational load characteristics of an application submitted to cloud computing are the most important factor. When the server is utilized to run numerous applications on the same platform simultaneously, it is overloaded. In virtual computers, to achieve effective resource utilization, a load balancer is used. The cloud uses a wide range of load-balancing techniques to enable effective control of the computational demand profile.

Incorporating a cloud–fog environment in SG-related processes requires an electricity load profile also called a computational load profile. During the implementation of the proposed model, each region is assigned a unique number of structures and fogs. These buildings are prevalent in a range of environments, including residential, commercial, and industrial situations. 

### 3.1. Problem Formulation

The proposed structure for the system consists of three levels, as illustrated in Figure 1. The top layer consists of clouds. The fog node is an intermediate layer. The third and last layer is the user layer. For ensuring the needs of the users are met, these levels communicate with each other. Through SG, the user asks the fog nodes to do calculations and other important tasks. The fog node layer is made up of virtual machines and microgrids. When a user approaches the fog node with a request for electricity, the fog nodes react by supplying the desired services by communicating with the nearest available resource that is capable of handling such requests. The fog node utilizes its resources effectively to satisfy the needs of its users. On the top layer, the Data Centers (DCs) are present housing *n* number of Physical Machines (PM). The set of DCs and PM is represented in Equation (1) and Equation (2), respectively.

DC = {DC_1_, DC_2_, DC_3_, … DC_n_} (1)

PM = {PM_1_, PM_2_, PM_3_, … PM_n_} (2)

In the proposed study, performance is evaluated based on parameters such as processing time, response time, and overall cost. The fog nodes are made up of virtual machines and microgrids. VMs manage varying numbers of concurrent processes. Using the mathematical notation, the set of virtual machines (VMs) and jobs (J) is illustrated in Equations (3) and (4).

VM = {VM_1_, VM_2_, VM_3_, … VM_n_} (3)

J = J_1_, J_2_, J_3_, … J_m_ (4)

In the proposed model, each VM operates concurrently. Jobs are submitted randomly and dynamically. Virtual machines must be flexible and adaptable due to the wide variety of tasks. Users’ requests are handled by each virtual machine according to processing and response time.

In the proposed work, the energy model is only used to figure out how much energy the cloud nodes need. The model does not account for the energy needed for smart grid applications, fog nodes, and cumulative energy consumption. The paper aims to reduce the time needed for processing time, response time and energy consumption. Thus, the problem is converted into a minimization problem of over-processing, response time, and energy efficiency. Therefore, the objective functions considered in the proposed work can be mathematically expressed as

(5) $$T_{min}=\sum_{i=1}^{n}\sum_{j=1}^{m}\left(RT*PT*DY\right)$$

(6) $$E\_i=Min(\sum_{i=1}^{m}\int_{{ST}_{time}}^{{FT}_{time}}E_{i}\left(U,F,T\right)$$

Equations (5) and (6) are the fitness function against which the performance of each iteration is measured.

### 3.2. Proposed Approach

The rock hyrax, known scientifically as Procavia capensis, is a small, furry mammal that inhabits rocky habitats throughout sub-Saharan Africa and along the Arabian Peninsula’s coast [15,38]. These mammals live in colonies of 50 members that are often dominated by a single male who protects the territory. They sleep, hunt for food, and even feed their offspring together. 

Every day, rock hyraxes feed circularly, with their heads turned away from the core of the circle so that they can keep an eye out for potential predators. When the group is feeding, either the breeding male or a female (the Leader) keeps a watch on a high rock or branch and will emit a shrill alarm or call if there is a potential threat, at which point, the group will run for cover.

Rock hyraxes hunt for food in groups. They have a very unique way of catching prey that involves forming a circle with different lengths and angles. When. When food is located, the Leader moves to a more elevated position so that they may both discover more food and defend each other from dangerous creatures. The paper utilizes a Rock Hyrax Optimization for balancing the load in a smart grid using Cloud and fog nodes. Like rock hyraxes, the proposed method checks the load on the virtual machine by employing the Divide and Conquer strategy and informing the aggregator about the state of virtual machines.

The proposed algorithm RHO comprises two different parts. Initially, RHO detects underutilized and over-utilized fog nodes. The algorithm moves the underutilized virtual machines to over-utilized nodes if a node is detected as underutilized during the process. The system considers all hosts other than the over-utilized host as underutilized hosts. The intention is to transfer the underutilized virtual machines and put such hosts into sleep mode. The transfer of underutilized VMs minimizes energy consumption because if underutilized virtual machines are active; they will continue to consume the same amount of energy even when not processing any requests. The next step is to migrate virtual machines (VMs) from an underutilized host. 

#### 3.2.1. Identification of Over-Utilized Fog Node

When a fog node is already busy, it is hard for it to meet the needs of new jobs. Because of this, the RT of fog nodes will keep going up until it reaches its maximum. The RHO has been implemented to address the issue and over-utilized fog nodes can be identified. RHO is a relatively new optimization technique that can be implemented rapidly since it employs the divide-and-conquer principle. The other algorithms are stuck in the problem of local minima and thus are a bit slow. The algorithm creates groups based on profit for the service provider. The users/organization yielding more profit or higher financial gain potential are given higher priority. The nodes of the high-priority profit group are examined first, and then other nodes are examined. The fog nodes are assessed and detected to determine whether they are over or underutilized.

#### 3.2.2. Identification of Under-Utilized Fog Node

All the fog nodes go through the process, and as many fog nodes as possible are turned off ensuring the number of active fog nodes goes down which eventually decreases the energy requirement.

An over-utilized VM receives too many requests simultaneously, which it cannot handle. This increases the response time of the machine. To handle this, a threshold value is used on the request queues of the nodes. The threshold value can be static or dynamic [39,40,41]. Because static thresholds are ineffective for changing loads, a dynamic threshold is used. If the number of pending requests on a node is greater than the threshold value, the node is categorized as overutilized. Similarly, if the number of pending requests is lesser than the threshold value, the node is categorized as an underutilized machine. Once all machines are categorized, the load-balancing process starts and the transfer of requests is initiated. This categorizing process continues over time as new jobs arrive.

A fog is underutilized when it does not get many requests and only has enough work to handle. In the study, a fog node that is not being used enough is termed underutilized. Having hosts that are not being used enough uses the same amount of energy as having fog that is not being used enough, therefore moving all virtual machines from underutilized fog nodes to other fog nodes and then turning off the original fog nodes.

#### 3.2.3. Selection Policy

When a fog node is fully utilized, its virtual machines are required to move to other fog nodes in such a way that other fog nodes do not become over-utilized. So, the selection must be done considering the load balance on fog nodes. If the selected node degrades performance, then it is not necessary to select it.

#### 3.2.4. Proposed Algorithm

The cloud and fog nodes handle many requests, and the performance of the system is improved by making good use of the available resources. In the paper, the requests are processed as per the performance of the VM in the fog nodes. For all of the requests to be handled correctly, they must be given to the right VMs. Load balancing techniques must be put in place first to get the result. 

**Figure 2:**
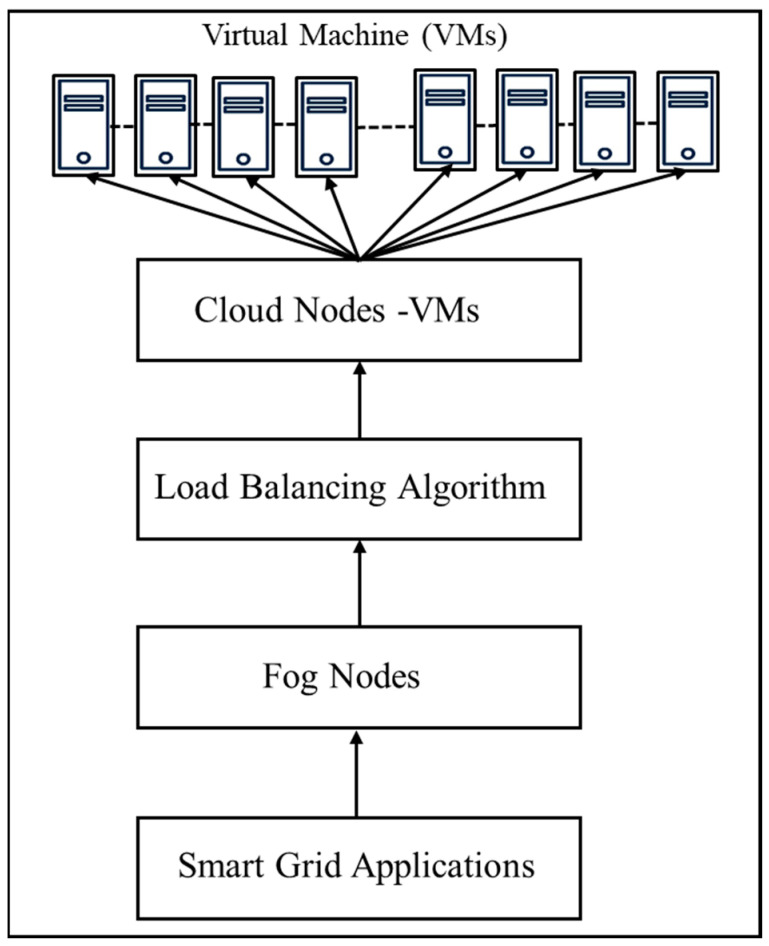
Load Balancing Process.

Figure 2 shows the process of load balancing. Users send requests to the person in charge of the fog node data center. The submitted requests are shared with the load balancer. The load balancer runs the load balancing algorithm to send requests to the right virtual machines. Virtual Machine Managers (VMM) manage VMs. The processing time, response time, and cost are analyzed for the proposed algorithm and compared to other algorithms like Throttled (Th) [42], Round Robin (RR) [43], Ant Colony Optimization (ACO) [32], Particle Swarm Optimization (PSO) [17].

Algorithm 1 takes the number of machines and requests them as input parameters, along with the threshold value. For every machine, as a new request is allocated, the queue length is increased by 1. The algorithm compares the value of the queue length with the threshold value. If the value of the queue length is lower than the threshold value, the machine is classified as an underutilized machine and called the Rock Hyrax Algorithm.
**Algorithm 1**: Algorithm to classify VM as Underutilized **Input**: Request *m*, Machine *n*, Threshold Value. **Result**: Servers *n_i_* having high load. Initialize *i, j* = 0; ∀ machine, **do** ∀ Request_m_ allocated to Machine_n_; QueueLength += 1; **end** **if** Queue length < Threshold **then**  Call RockHyrax Load Balancing Algorithm; **end**

An over-utilized VM receives too many requests simultaneously which it cannot handle. This increases the response time of the machine. To handle this, a threshold value is used on the request queue of the nodes. The threshold value can be static or dynamic. Static thresholds are not good for changing load; thus, a dynamic threshold is chosen. If the number of pending requests on a node is greater than the threshold value, the node is classified as overutilized. Similarly, if the number of pending requests is lesser than the threshold value the node will be declared as an underutilized machine. Once all machines are classified, the load-balancing process starts and the transfer of requests is initiated. This classification process continues over time as new jobs arrive.

Algorithm 2 takes in the number of virtual machines and requests and decides how busy the machines are. The VMs executes one task at a given time as allocated by the scheduler. The Rock Hyrax is represented as VM and the food is represented as jobs. The population of Rock Hyrax RH_num_ is initialized in the problem space. For every request, a VM that is the best fit for its execution is searched. The value of the fitness function is calculated for every Hyrax present in the environment. A variable Global_best_ is initialized to find the best fitness value. Until all the jobs and VMs are iterated and the number of iterations is not equal to the maximum iteration, the process will continue. For every iteration, the value of the fitness function is calculated. If the fitness function value is better than the value of Global_bes,_ it is updated else the fitness function is discarded. The Hyrax that has the best fitness value is declared Global_best_. The process continues until the value cannot be further improved. The result of the process is returned as Global_best_.
**Algorithm 2.** Rock Hyrax Load Balancing Algorithm**Input**: List of Request and VMs; Population ← InitializePopulation(RH_num_, Problem_size_); Initialize fitness function; Global_best_ ← 0; VM_best_ ← SelectBestVM(Population, VM_num_); **foreach** *VM_i_* ∈ VM_best_ **do**  SelectedRH_num_ ← ϕ;  Calculate the new fitness function;  iteration = iteration +1;  **If** Selected RH_num_ > RH_num_
**then**    RH_num_ ← Selected RH_num_;  **end if**  **Else**    RH_num_ ← RH_num_;  **end**  Calculate the fitness of chosen Hyrax; **If** Selected RH_num_ > RH_num_
**then**  Global_best_ ← RH_num_; **end for** **Return** Global_best_.

#### 3.2.5. Advantages of the Proposed Approach

The advantages of the proposed approach are:

Fog computing reduces cloud congestion and improves user communication. Wi-Fi connects users and fog nodes. Fog computing provides nearby services. Fog nodes receive user requests, and the VM’s many applications work together to meet them.

The transfer of underutilized virtual machines is done to minimize energy consumption because if underutilized virtual machines are active; they will continue to consume the same amount of energy even when not processing any requests.

RHO is a relatively new optimization method that uses the divide-and-conquer strategy and can be used quickly. The other algorithms are stuck in the problem of local maxima and thus are a bit slow.

The proposed algorithm is a multi-objective load-balancing optimization algorithm.

## 4. Performance Analysis and Discussion

The simulation software CloudAnalyst, which is an extended CloudSim simulator that simulates a real-world environment [44], has been used. Since all the computation is done on the cloud nodes, the performance of the proposed system will depend on the performance of the cloud nodes. For measuring performance, CloudAnalyst is used, which is built on top of the CloudSim toolkit, by extending its functionalities with the introduction of concepts that model Internet and Internet Application behavior. In the proposed network, four different geographical units were considered that represented the fog environment. Based on the load of the cloud nodes, the nodes are turned off to minimize energy consumption.

As the load-balancing algorithms can be either static or dynamic, the results of the proposed load-balancing algorithm are compared against static algorithms like Throttled, RR, and dynamic algorithms like ACO and PSO to ensure the validity of the results. In the considered model, two regions have four fog nodes. A certain number of PMs fall under the control of each fog node. Each of these groups contains multiple buildings. When a building or group of buildings requires electricity, the fog node that services them uses virtual machines to fulfil the demand. To evaluate the efficiency of various load-balancing strategies, experiments are carried out to compare response time, processing time, cost, and energy consumption. Static algorithms like throttled or Round Robin do not work well as the load request changes concerning time. The static algorithms require information about the load at a very initial state. So, the dynamic load declines their performance. Dynamic algorithms like ACO, PSO, and RHO can overcome the disadvantage of static algorithms. However, algorithms like ACO and PSO are stuck in local minima and are not able to explore all possible or the best solutions available. On the other hand, the proposed RHO works on the principle of divide and conquer and can explore and exploit the environment better than the other algorithms, providing better results. 

### 4.1. Experimental Setup

This section discusses the experimental setup carried out to evaluate the proposed method. CloudAnalyst [44] simulator running on Windows 7 was used to implement the proposed algorithm. The experimental setting is shown below. Table 3 shows the number of PMs considered in each fog node. 

Table 4 shows the physical capacity of each PM. Each PM is characterized by the number of processors and their speed, memory, and storage. Service providers can give users the option to use VMs instead of PMs thanks to virtualization technology.

Each physical machine has 20 virtual machines to handle the requests coming from the users. The configuration details of the VM are given in Table 5.

Through the smart grid application, users send their requests to the fog node to be carried out. The fog node, considering the load balancing algorithm, allocates the request to the virtual machine present in the cloud layer for execution. 

### 4.2. Response Time

The response time is calculated by subtracting the task’s start time from the user’s request time.

(7) $${RT}_{i}={DY}_{i}+{FT}_{i}-{ST}_{i}$$

where RT_i_ is the response time for Job_i_, DY_i_ is the delay in allocating resources to Job_i_, FT_i_ is the finish time of Job_i_, as well as ST_i_ is the submission time of Job_i_.

**Figure 3:**
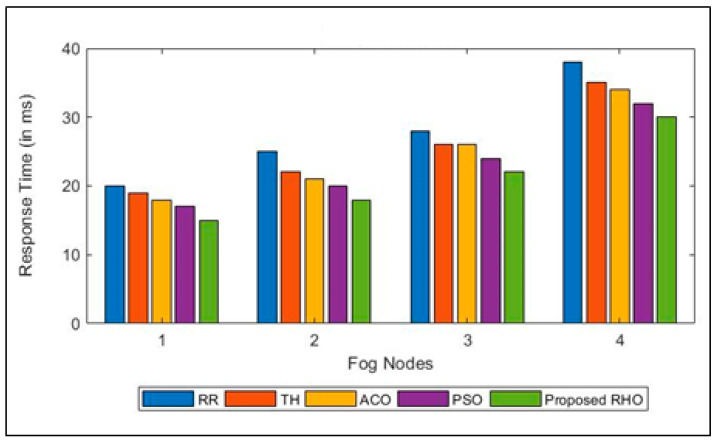
Response Time for different algorithms in different Fog Nodes.

**Figure 4:**
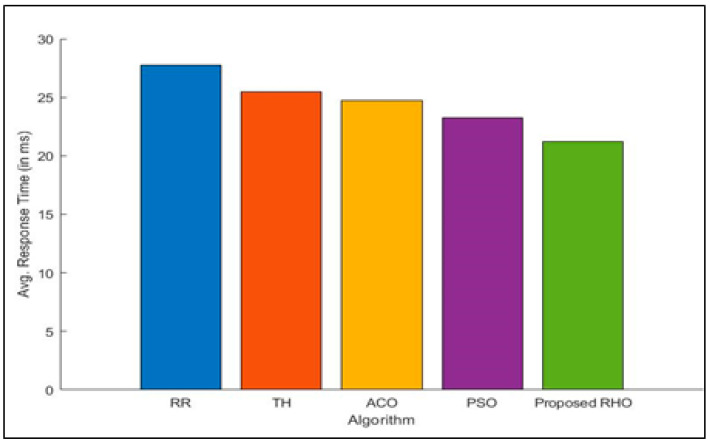
Average Response Time for different algorithms.

Figure 3 shows how long user groups take to respond when different algorithms are used. The response times for each user group were simultaneously optimized. The RHO facilitates the fog node work by allocating available resources efficiently. When a request is submitted to the fog node, the load balancer evaluates VMs to assess their capacity and energy consumption. In addition, the task at hand is given to the VM with the highest priority according to these numerous characteristics to assure that the job will not be delayed for an unreasonably long time. According to the experimental results, the proposed method is significantly more effective at load balancing. During peak hours, visibility may be quite low, but during off-peak hours, the fog node will move very quickly. The average response time for each fog node is shown in Figure 4 across the various methodologies. Results reveal that RHO can obtain a better average response time than popular algorithms described in the scientific literature. The proposed algorithm can improve the processing time by 23% over that of RR, 15% over Th, 14% over ACO, and 8% over PSO. The average improvement of the proposed algorithm is 15%.

### 4.3. Processing Time

The users submit a variety of jobs, each having a different processing length (PL) [27]. The amount of time required for processing is equal to the VM’s processing power (PP) and the length of the Job, as well as the magnitude of the task. Mathematically,

$$PT=\sum_{i=1}^{n}\sum_{j=1}^{m}\left({PP}_{i}*{PL}_{j}\right)$$

**Figure 5:**
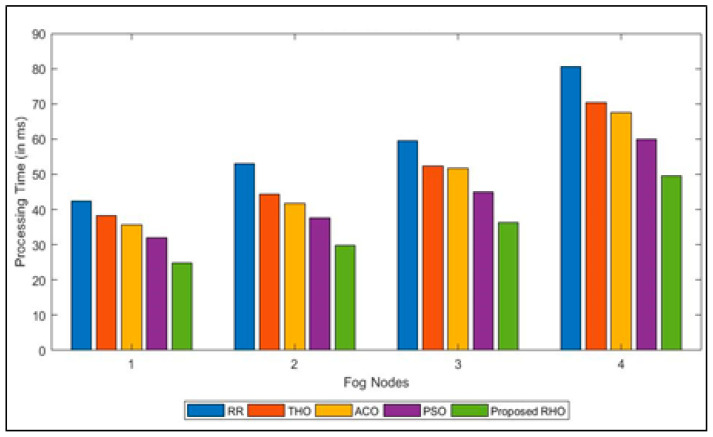
Processing Time for different algorithms for different Fog Nodes.

**Figure 6:**
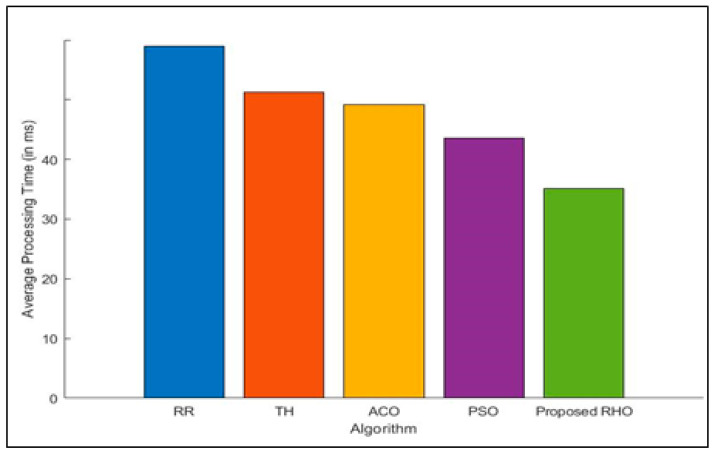
Average Processing Time for different algorithms.

Processing time for different algorithms to process jobs submitted by groups of users is illustrated in Figure 5. The figure depicts that RHO has a lower processing time than the other algorithm. The processing time for all users was optimized in parallel. With the help of many hyraxes, the proposed algorithm ensures that the load on the fog node is minimal. Using deterministic and random approaches the hyraxes try to find the best solution. The better performance could also be because the proposed algorithm has fewer coefficients that need to be tuned. Every time the process is repeated, the solution is optimized. The implementation of RHO keeps a single processor from getting too busy, which happens with other methods. Figure 6 shows the average processing time of a different algorithm. The proposed algorithm can improve the processing time by 37% over that of RR, 28% over Th, 26% over ACO, and 15% over PSO. The average improvement of the proposed algorithm is 26%.

### 4.4. Cost

Cost is another important parameter in Cloud and Fog. The cost depends on two things: how much it costs to move data and how much it costs to run a virtual machine. We have calculated different costs associated with the execution of the job [45,46]. These costs are VM cost (VM_cost_), the data transmission cost (DT_cost_), and the MG cost (MG_cost_) within the framework of the model that was presented. In the proposed model, the cost of the VM is calculated by considering both the number of MIPS and the size of the request.

(8) $${Cost}_{Total}={VM}_{Cost}+{DT}_{Cost}+{MG}_{cost}$$

The price of virtual machines (VMs) differs among fog nodes due to the disparity in the total number of VMs installed in each fog node. For simplification, let us assume the cost of utilizing the VM per hour is given by a constant value C. The VM_cost_ can be calculated as follows:

(9) $${VM}_{cost}=\left({VM}_{util}*{Toatl}_{VM}\right)*C$$

where VM_util_ is the time for which the VM was utilized. 

The MG_cost_ will be the cost running cost VM_i_ over MG_i_. Thus, mathematically can be expressed as

(10) $${MG}_{cost}={MG}_{i}*{VM}_{i}$$

DT_cost_ depends upon the data used and the cost of the transfer. Let the cost of transfer be given by Tc, for transferring data at VM_i_; then, DT_cost_ is expressed as:

(11) $${DT}_{cost}={DataUsed}_{i}*T_{c}$$

**Figure 7:**
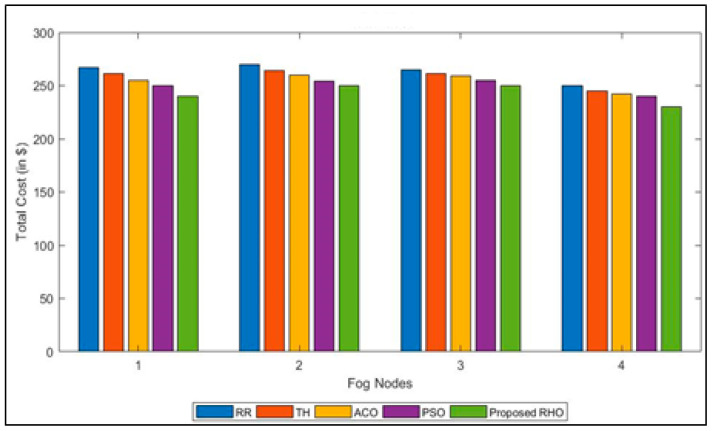
Total Cost for different algorithms for different Fog Nodes.

**Figure 8:**
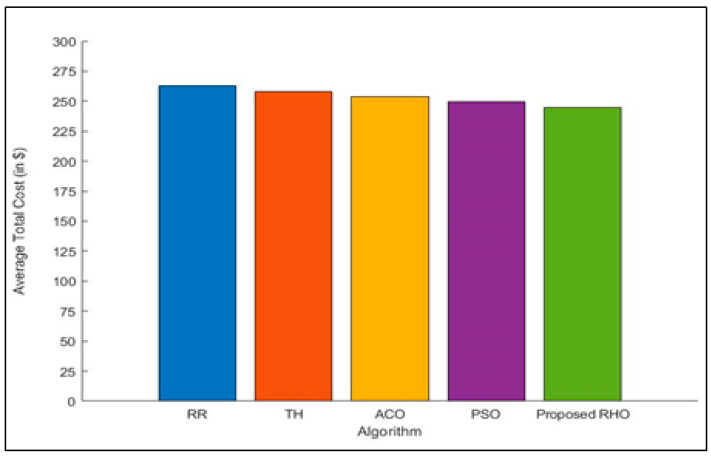
Average Total Cost for different algorithms.

Each fog node takes care of a lot of requests per hour [47,48]. The goal of the method is to keep costs as low as possible during peak times. There is a total of four different kinds of fog nodes in all the regions, and each has its cost component. In this section, all algorithms are used to compare the total costs for each of these parameters. Figure 7, which compares the proposed approach with the considered algorithm, shows the total cost of each fog node. When compared to other algorithms, the RHO usually has the lowest overall cost when it is used for most fog nodes. Figure 7 shows how much the round-robin, throttled, ACO, and PSO algorithms cost on average to make VMs when compared with the proposed RHO algorithm. Based on the simulation results, the proposed RHO algorithm works better than the considered algorithms. The improved performance of RHO comes from the fact that it can explore and exploit the environment better. RHO solves the problem on a global and regional scale. Figure 8 shows the average total cost of each fog node. The proposed algorithm can improve the cost by 9% over that of RR, 6.75% over Th, 5% over ACO, and 4% over PSO. The average improvement of the proposed algorithm is 6%.

### 4.5. Energy Efficiency 

The total power is needed by the data centers to manage physical machines and thus, to execute jobs in the cloud environment [30]. Mathematically, at a specific time t, the energy consumption by a PMi with a utilization factor UF is expressed as:

(12) $$E_{i}=\sum_{i=1}^{m}\int_{{ST}_{time}}^{{FT}_{time}}E_{i}\left(UF,T\right)$$

where E_t_ is the total amount of energy utilized by PMs, ST_time_ is the starting time of resource utilization, FT_time_ is the end time of resource utilization, and E_i_ is the amount of energy consumed by resource.

In the proposed work, the energy model is only used to figure out how much energy the cloud nodes need. The model does not consider the energy required by the SG applications, Fog nodes, and cumulative energy consumption.

**Figure 9:**
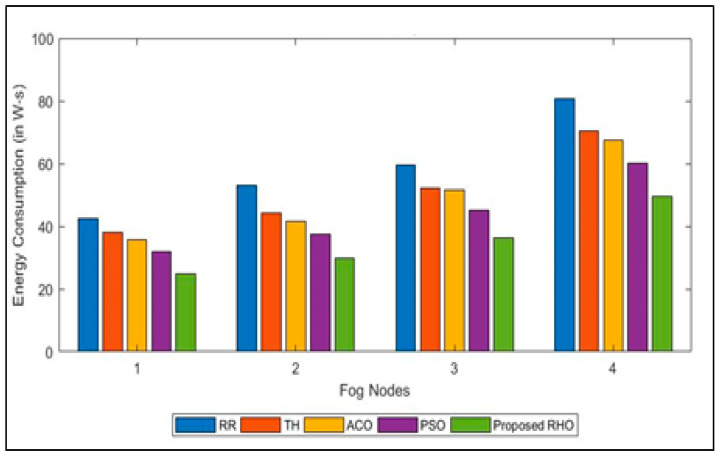
Energy Consumption for different algorithms for different Fog Nodes.

**Figure 10:**
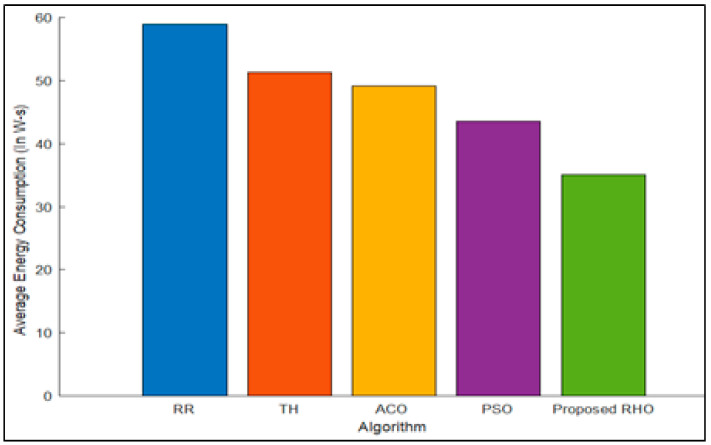
Average Energy Consumption for different algorithms.

The energy required to run various applications in SG connected to various devices is shown in Figure 9. The average energy consumption for the different algorithms is shown in Figure 10. The figure illustrates that the proposed algorithm requires less cost for the same number of applications across the different fog as the proposed algorithm finds the fog that is underutilized and migrates the resources from it to the fog that is overutilized while closing the resources. Turning off the underutilized fog nodes lowers energy consumption. The other algorithms use the resources even if the load on the fog is at a minimum, increasing the energy consumption. The proposed algorithm can improve the processing time by 40% over that of RR, 31% over Th, 28% over ACO, and 19% over PSO. The average improvement of the proposed algorithm is 29%.

### 4.6. Delay

The main reason for the delay is that the job is not executed in the allotted time. So, if we can lessen the amount of time it takes to finish a task, the total RT will also go down. In this work, the transfer time, and the latency time, which are two different kinds of time delays, are considered. Thus, the delay can be calculated as:

(13) $$DY={Transfer}_{Time}+{Latency}_{Time}$$

where latency delay refers to the amount of time it takes a virtual machine (VM) to perform the actions that a user instructs it to perform. The length of time required for a virtual machine (VM) to transmit a request made by a user to a server is referred to as the transfer time:

(14) TransferTime=DYBW

where BW is the available bandwidth, given by

(15) BW=BWTotalm

where BW_total_ is the total available bandwidth with the network, and M is the total number of submitted jobs.

**Figure 11:**
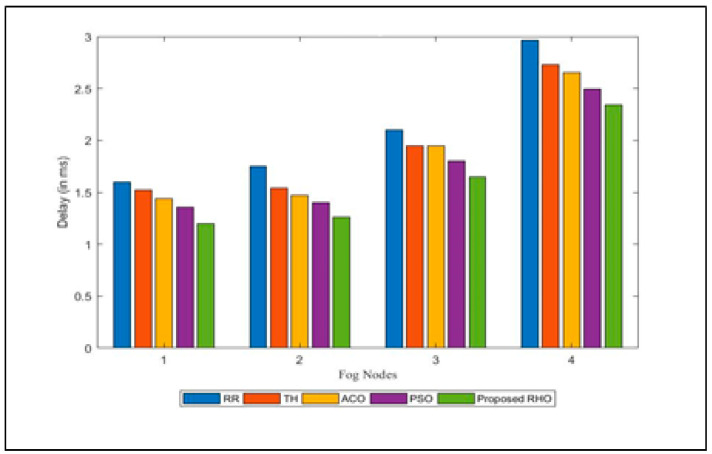
Delay for different algorithms for different Fogs Nodes.

**Figure 12:**
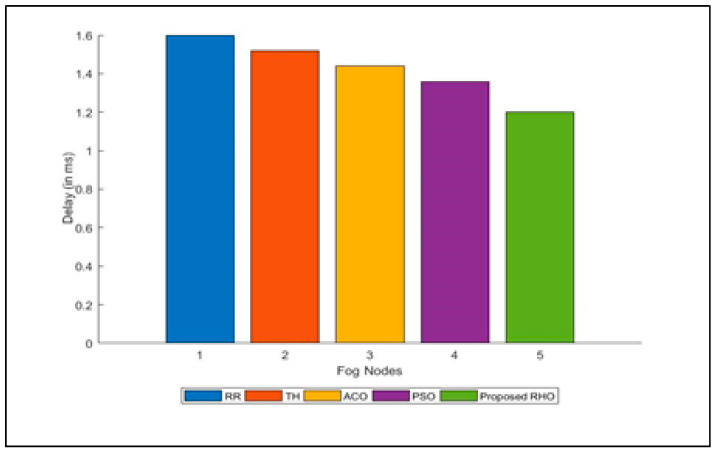
Average Delay for different algorithms.

The delay in execution of the request submitted to various fog is shown in Figure 11. The average delay for the different algorithms is shown in Figure 12. The figure illustrates that the proposed algorithm minimizes the delay in the execution of the job. Delay is caused when a request does not execute in the desired time. As the proposed algorithm minimizes the response time of the request, it automatically reduces the delay in the execution of the job. The proposed algorithm reduces processing time by 23% compared to RR, 17% compared to Th, 11% compared to ACO, and 6% compared to PSO. The average improvement of the proposed algorithm is 14%.

Table 6 shows the results of all QoS parameters for different algorithms that were found when different algorithms were run in CloudAnalyst. All results are for the average value obtained. 

### 4.7. Analysis of the RHO Algorithm

In the paper, a load-balancing approach for Smart Grid using Rock Hyrax Optimization (RHO) to optimize response time and energy consumption is presented. Let *m* represent the number of VMs and *n* the number of jobs. The algorithm works in two phases. In the first phase, it classifies the VMs as underutilized. The algorithm runs for the number of VMs in the environment; thus, the running complexity of phase I is ***O(m)***.

In the second phase, the load-balancing algorithm is executed. The algorithm runs until all jobs are not mapped. It searches for the best VM iteratively for all submitted jobs. Given that the for loop runs for the total number of jobs, the total number of operations is |m| × |n|. Thus, the complexity of the load-balancing algorithm is ***O(mn)***. Therefore, the overall complexity of the algorithm is ***O(m^2^n)***.

## 5. Conclusions

Every single day, more information is added to cloud storage. Users send requests to servers to get the energy they need. Because traditional grids are being changed into smart grids, there is a link between the people who use energy and the people who supply it. Since the number of users and requests is increasing, it is essential to find a method for the division of work. In the current study, a system model where SG is included in a cloud-fog environment is depicted. The model is divided into three layers: the cloud layer, the fog node layer, and the end user layer. The paper describes a method for load balancing that uses both cloud computing and fog computing to make sure that requests from users in residential areas are handled fairly. We used RHO to send user requests to different servers. The method manages load balancing while minimizing energy. With the help of CloudAnalyst, the simulations are run. The result of the proposed load-balancing algorithm is compared to several other static and dynamic methods. The results show that RHO works much better than the other algorithms because it works on the divide-and-conquer principle. The other algorithms are stuck in the problem of local maxima and thus, are a bit slow. In the future, the rock hyrax optimization can be used to exploit the multi-objectives, multi-dimensional knapsack problem.

## Figures and Tables

**Table 1:** AMI services with the corresponding layers.

Function	Service	Security	Latency	Layers
Control	Remote Access	High	Low	Fog & Cloud
	Demand Response	High	Medium	Fog & Cloud
	Remote Programming	High	Low	Cloud
	Feedback	High	Low	Fog
Billing	Time-Based	Low	Low	Fog & Cloud
	Automatic	High	Low	Cloud
Monitoring	Tamper detection	High	Low	Fog
	Power quality	High	Low	Fog & Cloud
	Outage notifications	High	Low	Fog & Cloud
Protection	Fault detection	High	Low	Fog
	Fault tolerance	High	Low	Fog
Reporting	Profiling	Medium	Low	Cloud

**Table 2:** Summary of Related Work.

Ref.	Objectives	Techniques	Achievements	Limitations	Performance Metric	Simulation Tool
[16]	Managing Energy by balancing the load	Dynamic Service Proximity	Nearest Fog that is best suitable to do the job	Cost is maximize	Energy Management	Cloud Analyst
[17]	Provision of computing resources for Smart Grid Management	Hybrid artificial bee ant colony optimization (HABACO)	Better efficiency while managing the load on SG	Energy is not considered	Response time	Cloud Analyst
[18]	Schdeuling of Job while balancing laod	Hybrid COA and PSO	Services on the cloud are scheduled	Unnoticed costs related to VMs	convergence speed	Matlab
[19]	Resource allocation by balancing load	Fog2-Cloud framework framework	Minimized response time	Static Algorithms compared	Response time and processing time	--
[20]	Switching VMs to a different location	Cuckoo optimization algorithm	Energy consumption reduce	Response time is not considered	Energy Management	Matlab
[21]	Load balancing through clustering	Artificial Bee Colony optimization	Reduction in makespan time	Only compared with non-clustered ABC	Makespan time	CloudSim
[22]	Load balancing by managing resources	Gradient Based Optimizer	Minimized response time	Energy is not considered	Response time	Python
[23]	Load balancing by managing resources	Hybrid WOA-BAT algorithm	Minimized response and processing time	Energy is not considered	Response time and Processing time	Cloud Analyst
[24]	Resource allocation to optimize resources	Particle Swarm Optimization with Simulated Annealing (PSOSA)	Minimized Cost	Maximum Response and processing time are higher	Cost	Cloud Analyst
[25]	Load balancing	Throttled load balancing algorithm	Efficient energy management of resources	Only static algorithms considered	Energy Management	Cloud Analyst

**Table 3:** Number of PMs in each Fog Node.

Fog Node	Number of PMs
1	20
2	15
3	10
4	5

**Table 4:** PM Characteristics.

Parameters	Values
Storage	1 Tb
Memory	128 GB
Bandwidth	1000 kHz
Number of Processors	4
Speed of Processor	10,000 MIPS

**Table 5:** VM Characteristics.

Parameters	Values
Storage	20 GB
Memory	4 GB
Policy	Time Sharing

**Table 6:** Comparison of Various QoS parameters obtained after Experiments.

Algorithm	RT	PT	Cost	Energy	Delay
RR	27.75	57.91325	263	58.91325	2.1
TH	25.5	50.306	257.75	51.306	1.93
ACO	24.75	49.17825	254	49.17825	1.8
PSO	23.25	42.617	249.75	43.617	1.7
Proposed RHO	21.25	36.1475	245	35.1475	1.6

## Data Availability

Not applicable.
